# Effects of Orally Administered Lactoferrin and Lactoperoxidase-Containing Tablets on Clinical and Bacteriological Profiles in Chronic Periodontitis Patients

**DOI:** 10.1155/2011/405139

**Published:** 2011-04-03

**Authors:** Eiju Shimizu, Tetsuo Kobayashi, Hiroyuki Wakabayashi, Koji Yamauchi, Keiji Iwatsuki, Hiromasa Yoshie

**Affiliations:** ^1^Division of Periodontology, Department of Oral Biological Science, Niigata University Graduate School of Medical and Dental Sciences, Niigata, Japan; ^2^Shimizu Dental Clinic, Takasaki, Japan; ^3^General Dentistry and Clinical Education Unit, Niigata University Medical and Dental Hospital, 2-5274 Gakkocho-dori, Chuo-ku, Niigata 951-8514, Japan; ^4^Food Science & Technology Institute, Morinaga Milk Industry Co., Ltd., Zama, Japan

## Abstract

This study was undertaken to evaluate the effect of oral administration of lactoferrin (LF) and lactoperoxidase-(LPO-)containing tablet on periodontal condition. Seventy-two individuals with chronic periodontitis were randomly assigned to take either bovine LF and LPO-containing tablets (test group, *n* = 37) or control tablets (control group, *n* = 35) every day for 12 weeks. Periodontal parameters and levels of subgingival plaque bacteria, human and bovine LF, and endotoxin in gingival crevicular fluid (GCF) were evaluated at baseline, 1 week, 4 weeks, and 12 weeks. Significant differences were observed in GCF levels of bovine LF between the test and control groups throughout the study (*P* < .05). However, clinical and bacteriological parameter values proved comparable between the two groups at 1 week to 12 weeks. Therefore, the effect of oral administration of LF and LPO-containing tablets might be weak on periodontal and bacteriological profile in this study.

## 1. Introduction

One of the essential components of therapy for periodontal diseases is elimination or control of periodontopathic bacteria. This has been accomplished predominantly by mechanical strategies including oral hygiene techniques and scaling/root plaining [1], occasionally being time consuming and ineffective in sites with difficulties of instrumentation [2]. Therefore, a variety of locally delivered antibacterial agents have been recommended as an adjunct method [3, 4]. However, these antibacterial agents were shown to originate some local side effects [3, 5], suggesting the need for antiinfective products to be used more safely. 

Lactoferrin (LF) is an 80-kDa iron-binding glycoprotein of the transferrin family and is a component of saliva as well as milk, tears, and secondary granules of neutrophils [6]. LF has been shown to have a diverse range of biological properties, for example, antibacterial, antiviral, and antioxidant activities [7, 8]. It has also been demonstrated that LF showed an in vitro antibacterial activity against periodontopathic bacteria, including *Porphyromonas gingivalis*, *Prevotella intermedia,* and *Aggregatibacter actinomycetemcomitans *[9–12]. Especially, the growth of *P. gingivalis* was strongly inhibited even with a low concentration (13.6 *μ*M) of LF [12]. Recently, our in vitro study indicated the inhibitory effects of bovine LF on biofilm formation of *P. gingivalis* and *P. intermedia *[13]. We also demonstrated that oral administration of bovine LF reduced the number of these periodontopathic bacteria in subgingival plaque of periodontitis patients [14]. 

Lactoperoxidase (LPO) is a member of the mammalian heme peroxidase family and is a component of saliva, milk, tears, and other exocrine secretions [15]. LPO catalyzes the hydrogen peroxide-dependent oxidation of thiocyanate to hypothiocyanate, exhibiting antimicrobial properties [15]. It has also been documented that growth and viability of oral bacteria were reduced by LPO thicyanate-hydrogen peroxide system [16, 17]. 

 Recently, LF and LPO-containing tablets were shown to exhibit a possible inhibitory effect on bacteria in saliva and oral malodor [18]. Therefore, we conducted a double-blinded, randomized, controlled trial in periodontitis patients to evaluate the efficacy of LF and LPO-containing tablets on periodontal parameters, and levels of subgingival plaque bacteria, and bovine and human LF, and endotoxin in gingival crevicular fluid (GCF). 

## 2. Methods

### 2.1. Subjects and Clinical Assessments

A total of seventy-six individuals who had been referred to Shimizu Dental Clinic were recruited between October 2008 and July 2009 for this study. Signed informed consent was obtained from all participants; the format of the study was reviewed and approved by the Research Ethics Committee of Niigata University Faculty of Dentistry (no. 20-R23-08-08, on August 25, 2008) in accordance with the Helsinki Declaration of 1975 and as revised in 2000. Clinical periodontal assessments were performed by one trained and calibrated examiner of the authors (E. S.) who were masked to the group assignments. The calibration was performed before the study with 5 volunteer subjects in Niigata University Faculty of Dentistry. The reproducibility of the clinical measurements was calculated by means of the *κ* index, and a value of 0.857 was obtained for clinical attachment level (CAL) with a difference of ±1 mm. All participants were clinically evaluated in the following periodontal measurements: number of teeth present, plaque index (PlI) [19], gingival index (GI) [20], probing depth (PD), CAL, plaque control record (PCR) [21], and bleeding on probing (BOP) [22]. PlI, GI, BOP, and PCR were measured at four sites around each tooth, whereas PD and CAL were assessed using a CP-12 probe (Hu-Friedy, Chicago, IL) at six sites around each tooth: mesio-buccal, mid-buccal, disto-buccal, mesio-lingual, mid-lingual, and disto-lingual. Measurements of PD were recorded to the nearest millimeter, and every observation close to 0.5 mm was rounded to the lower whole number. The mean value of each clinical parameter for each individual was used for the statistical analysis. The medical and dental records as well as smoking status of all participants were also checked with a standard questionnaire. Two subjects were excluded from this study according to the following exclusion criteria: (1) the presence of systemic disease (e.g., diabetes mellitus, kidney, liver, or lung disease), medication, and pregnancy, (2) having less than 20 teeth, (3) showing allergic reactions to cow's milk, (4) having antibiotic treatment within the previous 3 months, (5) having periodontal therapy within the previous 6 months, and (6) the current smoker. As a result, seventy-four subjects (33 males and 41 females, age range 32–73 years) with chronic periodontitis were selected for this study.

### 2.2. Study Tablets

The test tablet  (Morinaga Milk Industry Co., Ltd., Tokyo, Japan) contained 11.1% (weight/weight) LF, 0.2% LPO, 2.7% glucose oxidase, 3.0% glucose, 3.5% trisodium citrate dehydrate, 1.6% citric acid, 30.0% erythritol, 7.5% xylitol, 36.0% maltitol, 0.3% flavor, 0.2% l-menthol, 2.0% sucrose fatty acid ester, and 2.0% glycerol fatty acid ester. The composition of the control tablet (Morinaga Milk Industry Co., Ltd., Tokyo, Japan) was 83.9% maltitol, 11.1% cornstarch, 0.5% coloring materials, 0.3% flavor, 0.2% l-menthol, 2.0% sucrose fatty acid ester, and 2.0% glycerol fatty acid ester. LF (Morinaga Milk Industry Co., Ltd., Tokyo, Japan) and LPO (Biopole, Gembloux, Belgium) were purified from bovine milk, whereas glucose oxidase (Sumizyme PGO, Shin-Nihon Chemical, Aichi, Japan) was originated from *Penicillium chrysogenum*. These test and control tablets were round in shape with a 12-mm diameter and 6-mm thickness and were also identical in weight (900 mg), taste, texture, and appearance.

### 2.3. Study Protocol

**Figure 1:**
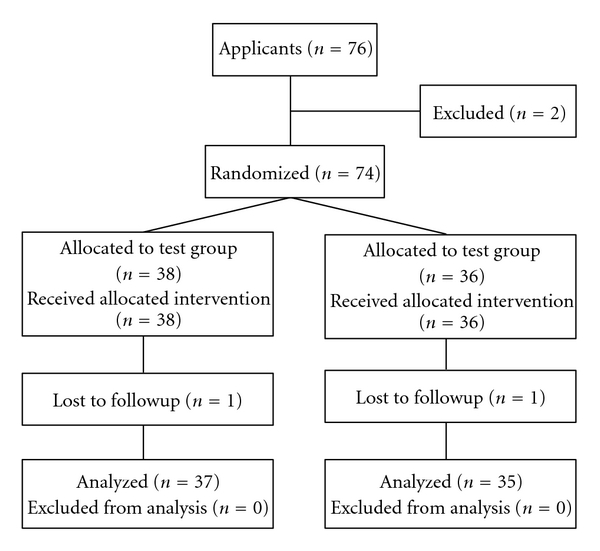
Flowchart of participants through each stage of the randomized trial.

This study was performed as a double-blinded, randomized, controlled design during 12 weeks (12 W). All individuals were randomly assigned to oral administration of either one tablet containing bovine LF (100.0 mg/tab) and bovine LPO (1.8 mg/tab) in the test group (*n* = 38) or one control tablet in the control group (*n* = 36), three times per day for 12 W. An independent study coordinator (Morinaga Milk Industry Co., Ltd., Tokyo, Japan) generated a random allocation sequence using a computer-generated random code and marked on packages containing test or control tablets with the subject numbers. The allocation sequence was concealed until interventions were completed. A 12-week ration of the test or control tablet was distributed at baseline. All subjects were directed to place one tablet in the oral cavity for 5 minutes after the main meal, allowing it to dissolve. In addition, they were instructed not to change their oral hygiene regimens, not to take any foods and drinks containing LF, and not to use mouthwash and dentifrice containing LF and LPO throughout the study period. Neither professional prophylaxis nor tooth-brushing instruction was performed during and before the experimental period. Periodontal clinical parameters and samples of saliva, subgingival plaque, and gingival crevicular fluid (GCF) were obtained from all subjects on baseline, 1 W, 4 W, and 12 W. On the examination day, all participants underwent clinical assessment and sampling at the same appointment time (10:00 to 11:00, 14:30 to 15:30, or 19:00 to 20:00), that is two or three hours later following main meal, tooth-brushing, and the intake of tablet. General condition was evaluated by interviewing at each time examination. Teeth, oral mucosa, and tongue were also examined visually. After the baseline examination, one subject in the control group did not undergo any examinations at 1 to 12 W, and another subject in the test group was lost at 12 W. Finally, 72 subjects (37 in the test group and 35 in the control group) were subjected to the clinical, bacteriological, and biochemical analysis. The flowchart of all participants through each stage of the clinical trial is shown in Figure 1.

### 2.4. Sample Collection

After clinical measurements, saliva, subgingival plaque and GCF samples were obtained. Whole saliva was collected into sterile 15-ml plastic tubes by paraffin chewing stimulation for 5 minutes [23]. Aliquots were made from saliva samples and stored at −20°C until used. Sampling sites of the selected diseased two teeth with a PD of 4 to 9 mm per subject were isolated with sterile cotton rolls. Supragingival plaque was removed with sterile curettes, without touching the marginal gingival. Subgingival plaque samples were then taken by inserting two sterile #40 paper points (DiaDent Group International, Burnaby, Canada) consecutively into the periodontal pocket for 10 seconds. The paper points were placed in sterile 3.6-ml plastic tubes and stored at −20°C until extraction of genomic DNA. After 5 minutes later of completion of subgingival plaque sampling, GCF was collected from the same sites with filter paper strips (Periopaper, Proflow Incorporated, Amityville, NY) [24]. The strips were placed into the pocket until mild resistance was sensed, and left in place for 30 seconds. Each strip was vortexed vigorously in 1 ml of 0.05 M Tris-HCl buffer (pH 7.5) containing 0.1% bovine serum albumin and 0.1% sodium azide and was kept at 4°C. The GCF sample was then centrifuged at 3, 000×g for 5 minutes, and the supernatant was stored at −80°C until assayed.

### 2.5. Microbiological Examination

A quantitative analysis of *P. gingivalis *and the total bacteria was done with the Invader PLUS method [25], which combines the polymerase chain reaction amplification and Invader detection. In brief, bacterial DNA was extracted from the stored samples of saliva and subgingival plaque using a DNA blood mini kit (QIAamp, Qiagen, Hilden, Germany). The primers for *P. gingivalis* were based on a region of the 16S ribosomal RNA sequences as follows: forward primer (5′-GCGCTCAACGTTCAGCCT-3′) reverse primer (5′-CACGAATTCCGCCTGCC-3′). The primary probe and Invader oligo were based on sequences in the amplified regions as follows: primary probe (5′-CGCGCCGAGGGGCAGTTTCAACGGC-3′) and Invader oligo (5′-GCCGCCGCTGAACTCAAGCCCT-3′). Likewise, the primers, probe and oligo for the total bacteria were designed as follows: forward primer (5′-GGATTCGCTAGTAATCG-3′) and reverse primer (5′-TACCTTGTTACGACTT-3′), primary probe (5′-CGCGCCGAGGCCGGGAACGTATTCACC-3′) and Invader oligo (5′-TGACGGGCGGTGTGTACAAGGCA-3′), respectively. The Invader PLUS reaction was performed in a 15-*μ*l mixture containing specific primers, 50 *μ*M deoxynucleotide triphosphate (d-NTP), 700 nM primary probe, 70 nM Invader oligo, 2.5 U Taq polymerase (AmpliTaq Stoffel fragment, Applied Biosystems, Foster city, CA), and Cleavase XI Invader core reagent kit (genomic DNA, TWT, Madison, WI). The reaction mixture was preheated at 50°C for 2 minutes, and two-step polymerase chain reaction was carried out for 35 cycles (95°C for 1 second and 63°C for 1 minute). Fluorescence values of carboxyfluorescein were measured at a wavelength of 485 nm (excitation) and 530 nm (emission). The limit of detection was determined with dilutions of the bacterial DNA, and the standard curves were made on the crossing point determined by fit point methods. The number of bacteria was calculated from the standard curves and expressed as Log_10_ copies/ml and Log_10_ copies/site for the saliva and subgingival plaque sample, respectively. As the detection limit for the bacterial count was 3Log_10_ (= 1000)/ml or site, the lowest value was indicated as 1000 for the count of samples under the limit. According to the detection limit, the number (percentage) of saliva and subgingival plaque samples at baseline in which *P. gingivalis* was detected proved 16 (43.2%) and 26 (35.1%) in the test group, and 14 (40.0%) and 25 (35.7%) in the control group, respectively. The quantitative analysis of *P. gingivalis* and the total bacteria was performed three times in each sample to confirm the reproducibility of the outcomes.

### 2.6. Measurement of LF and Endotoxin Levels

The concentrations of bovine LF in the saliva and GCF samples were determined by an sandwich enzyme-linked immunosorbent assay (ELISA) with antibovine LF polyclonal antibody and horseradish peroxide-conjugated antibovine LF antibody (Bethyl Laboratories, Inc., Montgomery, TX). Levels of human LF in the saliva and GCF samples were measured by a sandwich ELISA assay with an antihuman LF polyclonal antibody (Dakopatts, Glostrup, Denmark). The microtiter plates were read at a wavelength of 405 nm for both LF levels with an automated microplate reader (MTP-32, Corona Electric Co., Ltd., Hitachi, Japan). The concentration of bovine and human LF was expressed as *μ*g/ml for the saliva sample and ng/site for the GCF sample, respectively. Sensitivity of the bovine LF measurements was 10 ng/ml and those of the human LF measurements was 50 ng/ml.

Saliva and GCF levels of endotoxin were measured by the Limulus amebocyte lysate assay using a commercially available kit (Endospecy ES-24S Set, Seikagaku Biobusiness Corporation, Tokyo, Japan), according to the manufacturer's instruction. All reagents, pipette tips and microtiter plates were pyrogen free. The coloriometric changes reflecting endotoxin levels were measured at a wavelength of 540 nm using an automated microplate reader (MTP-32, Corona Electric Co., Ltd., Hitachi, Japan). The concentration of endotoxin was expressed as equivalent of USP standard unit (EU)/ml and EU/site for the saliva and GCF sample, respectively. The lower limit of detection was 0.002 EU/ml. Measurements of bovine and human LF and endotoxin were performed three times in each sample to confirm the reproducibility of the outcomes.

### 2.7. Statistical Analyses

The post-hoc sample size estimates/power calculation test which was based on the number of* P. gingivalis *in subgingival plaque revealed that more than 26 patients in each of the two groups would meet the statistical power, with the following assumptions: 5% of alpha level, 0.81 of anticipated effect size, 0.8 of statistical power level, in two-tailed hypothesis. Differences between the test and control groups in demographic and clinical parameter values were assessed by Mann-Whitney *U* test, and by chi-square or Fisher's exact test for categorical variables, while those in bacterial counts and levels of endotoxin and LF were evaluated by Student's *t*-test. The unit of analysis was individual in demographic parameters (age, gender, smoking status, and the number of teeth present), PCR, the number of total bacteria and *P. gingivalis* in saliva, saliva levels of bovine and human LF and endotoxin, while that was site in PlI, GI, BOP, PD, CAL, the number of total bacteria and *P. gingivalis* in subgingival plaque, and GCF levels of bovine and human LF and endotoxin. All outcome analyses were performed according to the intention-to-treat principle. Statistical significance was set at *P* < .05. 

## 3. Results

**Figure 2:**
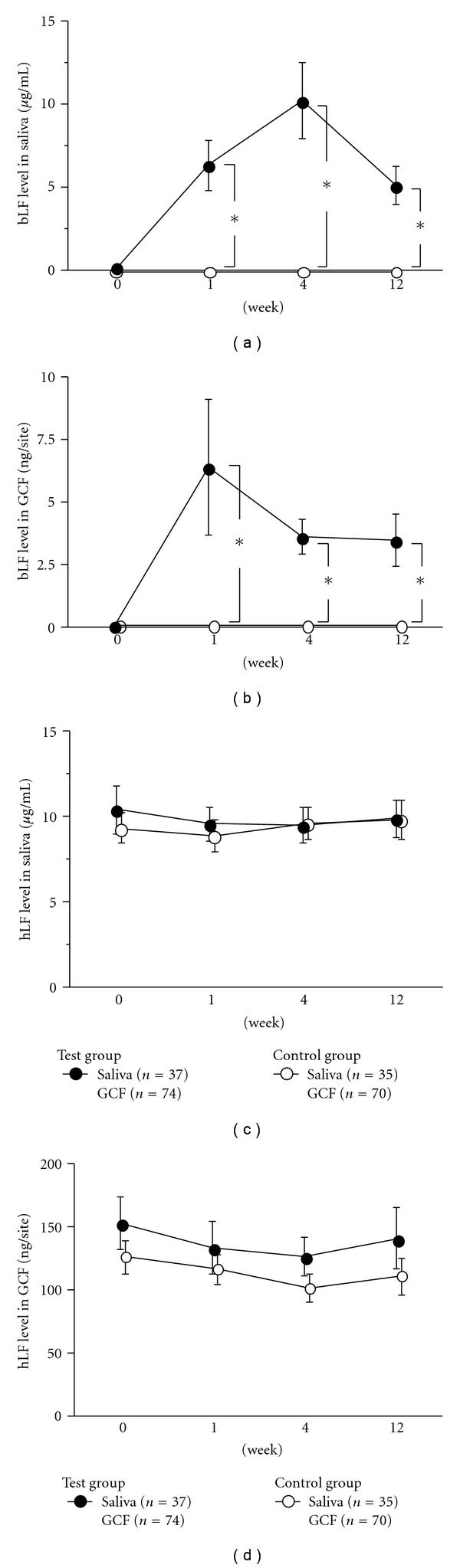
Effect of the test tablet on bovine and human LF levels in saliva and gingival crevicular fluid (GCF). Saliva levels of bovine LF (a), GCF levels of bovine LF (b), Saliva levels of human LF (c), and GCF levels of human LF (d) were measured at baseline, 1 W, 4 W, 12 W. Values represent the mean ± standard error. GCF levels of bovine and human LF were assessed in two teeth with chronic periodontitis per subject. *Significant difference between the test and control groups, as identified by Student's *t*-test (*P* < .05).

At baseline, we found no significant differences in any demographic and clinical periodontal parameter values between the test and control groups (Tables 1 and 2). Bovine LF levels in saliva and GCF in the test group were significantly higher than those in the control group (*P* < .05) (Figures 2(a) and 2(b)). However, no significant differences were observed in any clinical periodontal parameters at 1 W to 12 W between the two groups (Table 2). Human LF levels in saliva and GCF were also comparable between the two groups (Figures 2(c) and 2(d)). 

**Figure 3:**
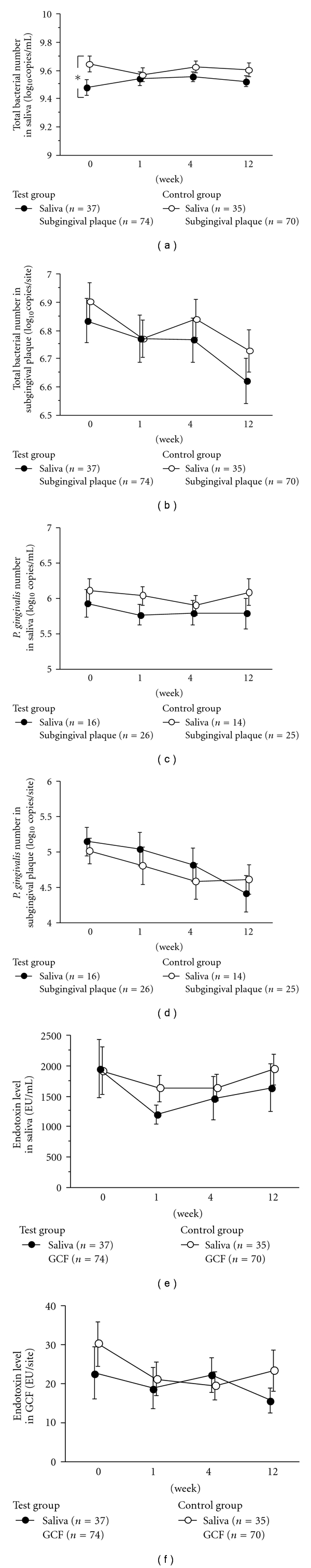
Effects of the test tablet on bacterial number and endotoxin levels in saliva, subgingival plaque, and gingival crevicular fluid (GCF). Total bacterial number in saliva (a), total bacterial number in subgingival plaque (b), *P. gingivalis* number in saliva (c), *P. gingivalis* number in subgingival plaque (d), saliva levels of endotoxin (e), and GCF levels of endotoxin (f) were measured at baseline, 1 W, 4 W, 12 W. Values represent the mean ± standard error. The number of total bacteria and* P. gingivalis *in subgingival plaque and GCF levels of endotoxin were assessed in two teeth with chronic periodontitis per subject.*Significant difference between the test and control groups, as identified by Student's *t*-test (*P* < .05).

The number of total bacteria and *P. gingivalis* in saliva and subgingival plaque was not significantly different between the two groups (Figures 3(a) to 3(d)), with exception that a significant intergroup difference in the number of total bacteria in saliva at baseline (*P* < .05). Endotoxin levels also proved comparable between the two groups throughout a 12 W-observation period (Figures 3(e) and 3(f)).

General condition was obtained by interviewing subjects at each time examination. No adverse events were observed in all participants during the study period. One subject in the control group was withdrawn at 1 W, and another subject in the test group was lost at 12 W. These subjects were also confirmed to exhibit no general symptoms before the withdrawal. Furthermore, abnormal effects on teeth, gingiva, oral mucosa, and tongue were not observed on visual examination.

## 4. Discussion

In this clinical trial, a total of seventy-two participants (37 in the test group and 35 in the control group) was subjected to the clinical, bacteriological, and biochemical analysis. Based on the reported changes in the number of *P. gingivalis *in subgingival plaque in the test and control groups ([mean ± SD]: 1.04 ± 1.72 and −0.20 ± 1.29 [log_10_/paperpoint] in our previous study [14], the number of subjects needed per the group was calculated by post-hoc sample size estimates/power calculation test, using the following assumptions: 5% of alpha level, and 0.81 of anticipated effect size, 0.8 of statistical power level, in two-tailed hypothesis. It was observed that more than 26 patients in each of the two groups, when randomly allocated, would meet the statistical power. 

The results of this study failed to demonstrate an oral administration of the test tablet containing bovine LF and LPO to be effective in improvements of clinical and bacteriological parameter values, although the test group showed a significant reduction in change in PlI score at 1 W, as compared to the control group (data not shown). These results were different from the findings of other studies indicating an in vitro antibiofilm activity of bovine LF [13], and antiinflammatory effects of LF and LPO [26, 27]. These observations might be partly attributable to insufficient concentration of LF for the antibacterial activity. The GCF concentrations of bovine LF in the test group were calculated to be 1.8 to 3.2 *μ*g/ml, on the basis of assumption that 2 *μ*l of GCF was obtained [24]. These LF levels were shown to be under the concentration of 8 to 31 *μ*g/ml, at which the antibiofilm activity of bovine LF proved effective against *P. gingivalis *[13]. Therefore, it is suggested that oral administration of tablet containing bovine LF (100.0 mg/tab) and bovine LPO (1.8 mg/tab) in this study would neither clearly influence the clinical periodontal condition nor the number of bacteria in saliva and subgingival plaque. Recent study documented that the same composition of bovine LF and LPO showed little effect on the numbers of some periodontopathic bacteria in saliva by quantitative polymerase chain reactions, which is in accordance with our results, but terminal restriction fragment length polymorphism (T-RFLP) detected one fragment with a significant lower number of the copy in the test group compared to that in the control group [18]. Thus, comprehensive T-RFLP analysis of bacteria in the plaque would be interesting in the future study.

Both test and control groups showed a weak trend toward a decrease in levels of periodontal inflammation (GI and BOP) and destruction (PD and CAL) and in the number of total bacteria and *P. gingivalis* in subgingival plaque. These observations might be partly explained by the antimicrobial activity of fatty acid esters of sucrose and glycerol [28, 29], which were added as lubricants to both the test and control tablet. Another possible explanation relates to an attention bias (Hawthorne effect) occurred within subjects in this study [30, 31]. Our results indicated that PCR scores and total bacterial counts had a tendency to decrease in both groups, although the experimental protocol did not include any oral hygiene instruction before or at baseline. It has been documented that many factors including attention bias contributed to perceived placebo effects in large-scale, randomized, controlled trials [31]. Furthermore, a recent study suggested that oral administration of the tablet itself may stimulate secretion of saliva, possibly affecting periodontal condition [32].

Apart from bovine LF and LPO, there were also differences in the contents between the test and control tablet. Glucose oxidase and glucose were added in the test tablet, leading to generation of hydrogen peroxide necessary for the LPO system [33]. Likewise, trisodium citrate dehydrate and citric acid were included as buffer salts into the test tablet to make the solvent a slightly acid, resulting in an optimal condition for the enzymatic activity of LPO. The test tablet also contains sugar alcohols such as xylitol and erythritol. These sugar alcohols were reported to exhibit a potential suppressive effect on biofilm formation in vitro [34–36], implicating some effects on periodontal condition. However, the concentrations of these sugar alcohols used for the test tablet were much lower than those reported in the studies [29, 37, 38]. Therefore, it is conceivable that the aforementioned components of the tablet would not influence clinical and bacteriological profiles. 

We performed both the individual and site analysis in this study, for better understanding of the efficacy of the test tablets on periodontal parameters, levels of subgingival plaque bacteria, and bovine and human LF and endotoxin in gingival crevicular fluid (GCF) in the periodontally diseased site. However, the site analysis was performed in either the individual site or tooth chosen, which may be influenced by the patient's individual characteristics. Therefore, it might be necessary when interpreting our results with the caution.

 LF and LPO are component of saliva, and were purified from bovine milk for the test tablet. Other components of the test tablet are also safe in health. All of these compositions have been permitted to be included in commercially available food and drink in Japan. Our results demonstrated that oral administration of these tablets showed no abnormal adverse events in general and oral condition. Therefore, it is expected that bovine LF and LPO-containing tablet would be used more safely, rather than the commercially available antiinfective products.

## 5. Conclusions

The results of our clinical study suggest that the effect of oral administration of LF and LPO-containing tablets might be weak on periodontal and bacteriological profile.

## Figures and Tables

**Table 1:** Demographic characteristics of participants.

Characteristics	Test group	Control group	*P*-value
Subjects (number)	37	35	
Age (mean ± SD years)	52.5 ± 11.8	51.7 ± 11.3	.71
Gender (number of Female/Male)	12/25	15/20	.06
Smoking status			.47
Current smoker (number)	0	0	
Former smoker (number)	3	5	
Never smoked (number)	34	30	
Number of teeth examined*	74	70	.22
Anterior teeth (number)	5	10	
Premolar teeth (number)	53	50	
Molar teeth (number)	16	10	

*Two teeth with chronic periodontitis were examined per subject for clinical, bacteriological and biochemical parameters.

Differences in age and gender between the two groups were assessed by Mann-Whitney *U* test, and those in smoking status and the number of teeth examined between the groups were evaluated by chi-square or Fisher's exact test, respectively.

**Table 2:** Effects of bovine LF and LPO-containing tablets on clinical periodontal parameters.

Parameter	Group (*n*)	Baseline	1 Week	4 Weeks	12 Weeks
PCR	Test (37)	58.33 ± 2.15	55.14 ± 1.80	52.36 ± 1.94	50.53 ± 2.29
	Control (35)	58.70 ± 2.09	58.43 ± 1.99	54.69 ± 2.40	53.69 ± 2.50

PlI	Test (74)	1.12 ± 0.04	1.01 ± 0.01	1.03 ± 0.03	1.04 ± 0.04
	Control (70)	1.04 ± 0.02	1.07 ± 0.03	1.01 ± 0.03	1.06 ± 0.03

GI	Test (74)	1.54 ± 0.06	1.39 ± 0.06	1.41 ± 0.06	1.34 ± 0.06
	Control (70)	1.47 ± 0.06	1.30 ± 0.06	1.37 ± 0.06	1.34 ± 0.06

BOP	Test (74)	56.76 ± 5.80	40.54 ± 5.75	40.54 ± 5.75	36.49 ± 5.63
	Control (70)	50.00 ± 6.02	32.86 ± 5.65	41.43 ± 5.93	34.29 ± 5.71

PD	Test (74)	4.97 ± 0.14	4.69 ± 0.11	4.58 ± 0.10	4.16 ± 0.09
	Control (70)	4.60 ± 0.09	4.49 ± 0.09	4.36 ± 0.07	4.04 ± 0.09

CAL	Test (74)	4.97 ± 0.14	4.69 ± 0.11	4.58 ± 0.10	4.18 ± 0.09
	Control (70)	4.60 ± 0.09	4.49 ± 0.09	4.36 ± 0.07	4.04 ± 0.09

Values represent the mean ± standard error.

PCR: plaque control record (%). PlI: plaque index. GI: gingival index. BOP: bleeding on probing (%). PD: probing depth (mm). CAL: clinical attachment level (mm).

PlI, GI, BOP, PD, and CAL were assessed in two teeth with chronic periodontitis per subject.
